# Supplementary material for: Evidence of validity and reliability of Jumpo 2 and MyJump 2 for estimating vertical jump variables
Source: PeerJ. 2023 Jan 25;11:e14558. doi: 10.7717/peerj.14558 (PMC9884043; doi:10.7717/peerj.14558)
Supplement: Supplemental Information 1 — Data are presented as mean and 90% of confidence limits (CL). [file peerj-11-14558-s001.docx]

Supplemental Table 1.⎯ Intra-rater reliability of measures obtained during the countermovement and squat jumps.

|  | **Countermovement Jump** | | | **Squat Jump** | | |
| --- | --- | --- | --- | --- | --- | --- |
| **Variables** | **Force Plate**  **CL 90%** | **Jumpo CL 90%** | **MyJump CL 90%** | **Force Plate CL 90%** | **Jumpo CL 90%** | **MyJump CL 90%** |
| Jump height (cm) | 0.97 | 0.94 | 0.98 | 0.94 | 0.95 | 0.92 |
|  | 0.93-0.99 | 0.78-0.98 | 0.94-0.99 | 0.82-0.98 | 0.85-0.98 | 0.76-0.97 |
| Mean force (N) | 0.96 | 0.98 | 0.99 | 0.98 | 0.99 | 0.96 |
|  | 0.87-0.99 | 0.99-0.94 | 0.98-1.00 | 0.94-0.99 | 0.99-1.00 | 0.88-0.99 |
| Mean velocity (m·s^-1^) | 0.96 | 0.85 | 0.97 | 0.84 | 0.96 | 0.92 |
|  | 0.88-0.99 | 0.60-0.95 | 0.90-0.99 | 0.60-0.95 | 0.88-0.99 | 0.76-0.97 |
| Mean power (W) | 0.94 | 0.93 | 0.98 | 0.93 | 0.97 | 0.92 |
|  | 0.83-0.98 | 0.80-0.98 | 0.93-0.99 | 0.80-0.98 | 0.91-0.99 | 0.76-0.97 |

Data are presented as mean and 90% of confidence limits (CL).
